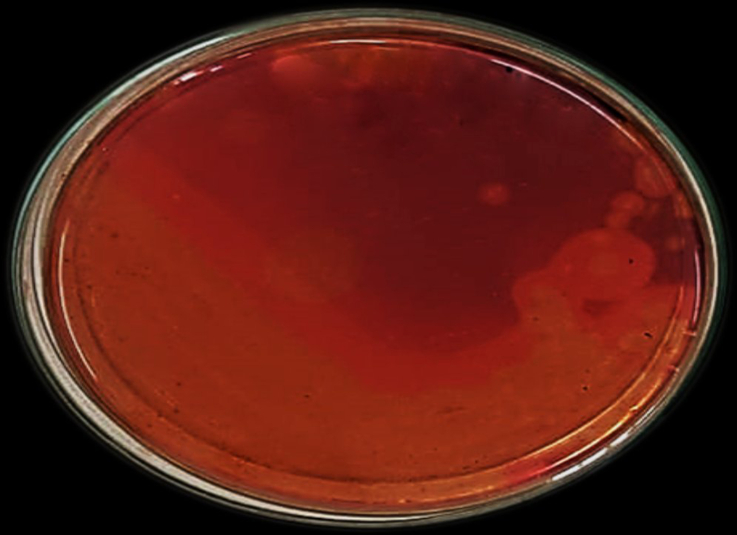# Valorization of rice stubble through biodegradation using hydrolytic enzyme-producing *Olivibacter oleidegradans* CMB10 and *Agrobacterium pusense* SFMB9

**DOI:** 10.1016/j.heliyon.2025.e42094

**Published:** 2025-01-17

**Authors:** Milind Gajbhiye, Sushmita Patil, Sagar Awate, Supriya Kokare, Siddharth Terdale, Manish Kumar Dubey

**Affiliations:** a Department of Microbiology and Research Centre, Tuljaram Chaturchand College, Baramati, 413102, Maharashtra, India; b Department of Biotechnology, University Centre for Research and Development, Chandigarh University, Chandigarh, 140413, Punjab, India

**Keywords:** Biodegradation, Stubble degradation, *Olivibacter*, *Agrobacterium*, Cellulase, Aerobic composting

## Abstract

Hydrolytic enzymes (cellulase, pectinase, xylanase) producing bacteria were isolated from compost, garden soil and wastewater. Out of 63 bacterial isolates, CMB10 and SFMB9, were selected for study due to their enzymatic potential, with relative enzyme activity ranging from 0.5 to 0.8. Both were Gram-negative, rod-shaped and non-motile. They were phenotypically characterized concerning Bergey's Manual of Determinative Bacteriology followed by molecular characterization by 16S rRNA gene sequencing and CMB10 and SFMB9 were identified as *Olivibacter oleidegradans* and *Agrobacterium pusense*, respectively. The two isolates were tested *in-vitro* for rice stubble degradation. The highest stubble degradation (86.3 %) was recorded in the case of co-inoculated treatment on 45^th^ day as compared with individual microbial treatments (*P* < 0.05). However, there was a significant difference between all the bacterial treatments and uninoculated control on 15, 30 and 45^th^ day of incubation. Scanning electron microscopic images confirm the distortion of microfibrillar structure of stubble by cultures. The activity of all hydrolytic enzymes was detected on all the days post-inocualtion, where, cellulase activity was at the highest (95.77 U/ml). There was an effective decrease in C/N ratio in all inoculated trials from 26.4 to 14.2 in 45 days of composting. This compost had a positive stimulatory effect on the growth of *Glycin max*, evidenced by ≈ 10 % increase in chlorophyll content of leaves, and ≈25–45 % increase in root and shoot length, respectively, as compared to uninoculated control. The isolates reported here for the first time concerning rice stubble degradation, show potential for large-scale stubble degradation.

## Highlights

• Biodegradation of rice stubble waste using bacterial consortium.
• First report of use of *Agrobacterium* sp. and *Olivibacter oleidegradans* in stubble valorization.
• *Agrobacterium* sp. and *Olivibacter* sp. produce a bouquet of hydrolytic enzymes.
• A straightforward and inexpensive approach to recycling of C, N and minerals in soil.
• Aerobic composting of rice stubble discourages farmers from burning residues.

## Graphical abstract

**Figure:**
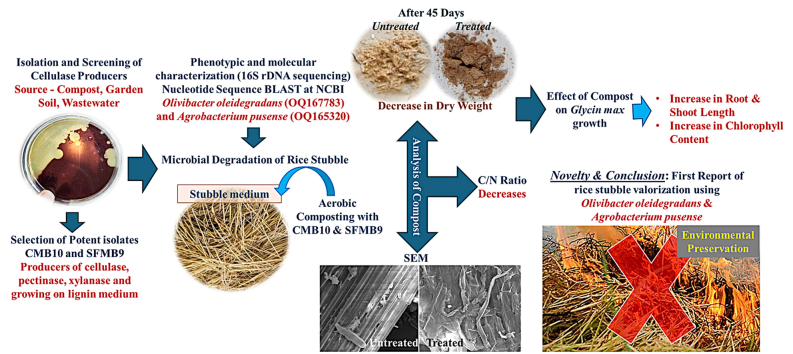

## 1 Introduction

Stubble is the straw and crown of plants left on the soil surface after harvest. Rice (*Oryza sativa*) is one of the important cereals used primarily for human consumption. It is a common practice of farmers worldwide to eliminate rice straw residue or trash through open burning in fields. This activity generates huge smoke and massive clouds of smoke streaking across the area negatively impacting the environment and public health. Concerning the annual report for year 2021 published by the Indian Agricultural Research Institute, New Delhi, India, the Indian states viz., Punjab, Haryana, Delhi & neighboring states, and West Bengal generate a huge amount of straw waste during the month of October and November each year. It was estimated that about 500 metric tons of stubble is generated each year in India, of which approximately 25 % is wheat stubble and 38 % is rice stubble. In general, farmers adopt the practice of burning it as a method of disposal. About 24 % of stubble is burnt on the field after harvest [1]. Burning stubble also affects soil fertility as many of the nutritive components such as organic and inorganic compounds (NPK) are lost from the soil [2]. Also, the dried top soil may be lost due to erosion. The population of plant growth promoting microorganism declines rapidly due to the burning effect [3].

Open burning of straw residues contributes significantly to air pollution as this activity causes the emission of gases such as CO_2_, CH_4_, CO, NO_2_ and SO_2_. Straw burning also releases aerosol particles that causes allergic reactions, respiratory and cardiorespiratory problems in pregnant women, elderly people, children with existing health issues such as asthma, bronchitis, tuberculosis, pneumoconiosis and other pulmonary infections [4,5]. Thus, stubble burning has become a severe problem in India as it causes several environmental and health problems [6]. Furthermore, many times, the rice fields are wetted after harvest, that may hinder the proper decomposition of straw and may lead to generation of unpleasant odour [7]. Straw from the fields can be used for several purposes that include construction materials, paper production, mushroom cultivation, and poultry litter. However, removing straw from fields may be unaffordable for farmers [8]. Considering the negative impact of stubble burning, there is a need for an eco-friendly solution for the management of stubble waste [7]. Unlike most cereals, rice straw, composed of 38.3 % of cellulose, 31.6 % of hemicellulose, 11.8 % of lignin, and 20 % of silica is resistant to bacterial decomposition due to its poor wettability. However, it can still be incorporated into soil [9]. Efforts have been made to improve the nutrient level in the soil to enhance productivity through the inclusion of stubble into the soil. This results in increased soil fertility and soil organic content maintenance. The biological approach of stubble degradation results in the valorization of this waste for use in the agriculture and fermentation industry. The bio-decomposition of stubble by microorganisms leads to the production of compost which is rich in organic and inorganic nutrients that improve the productivity of soil crops [10]. Thus, microbial degradation of stubble promotes nutrient recycling, and improvement in soil organic carbon and microbial biomass. This approach shall be useful in minimizing the pollution effects of stubble burning. Microbes such as bacteria, actinomycetes and fungi have been effectively used in the composting of rice stubble [11]. In this view, the major objectives of the present work were to isolate microbial producers of plant cell wall degrading enzymes (CWDE), characterize potent isolates, and evaluate their potential in the management of paddy straw, and its valorization.

## 2 Material and methods

### 2.1 Enrichment and isolation of cellulolytic bacteria

The samples such as compost, garden soil and wastewater were used for the enrichment of cellulase-producing bacteria in Carboxyl methyl cellulose (CMC) broth. CMC is a derivative of cellulose which is soluble in water and generally used for the detection of cellulose degraders. The inoculated flasks were incubated at 30 °C for 48 h under shaking conditions. For isolation, enriched broth was streaked on CMC agar followed by incubation. The bacterial cultures were maintained on nutrient agar slants at 4 °C for further use. Further, the cellulose-degrading ability of bacterial cultures was confirmed by spot inoculation of 10 μl of bacterial cell suspension on CMC (1 % w/v) agar, followed by incubation at 30 °C for 24 h and addition of 50 mM iodine solution. The clear zone formed around the bacterial growth was measured and relative enzyme activity was determined [12].

### 2.2 Screening for production of hydrolytic enzymes

The cellulolytic bacterial isolates were further screened for their enzymatic degradation ability of different polymers by determining the production of amylase, pectinase and xylanase. Briefly, 10 μl of bacterial cell suspension was added to the centre of starch agar, pectin agar and xylan agar separately for the detection of amylase, pectinase and xylanase activity. After incubation, the clear zones visible after the addition of iodine solution were measured, and relative enzyme activity was determined as above. The lignin degradation ability of bacterial cultures was determined as described by Kausar et al. [13]. Briefly, bacteria were streaked on a solid medium containing azure B (0.01 % w/v) followed by incubation and observation of plates for the growth of bacterial colonies.

### 2.3 Phenotypic and molecular characterization of potent degraders

The selected bacterial strains were culturally characterized by detecting colony characters on nutrient agar, Gram's staining and KOH test. Further biochemical characterization was done using guidelines of Bergey's Manual of Determinative Bacteriology. Molecular characterization of the bacterial isolates was done by 16S rRNA gene sequencing. Briefly, the genomic DNA was isolated by standard phenol/chloroform extraction method followed by PCR amplification of the 16S rRNA gene using universal primers 16F27 [5′-CCAGAGTTTGATCMTGGCTCAG-3] and 16R1492 [5′TACGGYTAC CTTGTTACGACTT-3]. The amplified 16S rRNA gene PCR product was purified by PEG-NaCl precipitation and directly sequenced on an ABI®3730XL automated DNA sequencer as per the manufacturer's instructions. Essentially, sequencing was carried out from both ends using additional internal primers so that each position was read at least twice. Assembly was carried out by using Laser gene packaging followed by identification using the EzBioCloud database. A phylogenetic tree was constructed by Neighbour-Joining tree method using MEGA X. The nucleotide sequences of 16S rDNA were deposited in NCBI GenBank database and accession numbers were obtained.

### 2.4 In vitro management of rice stubble

#### 2.4.1 Processing of the rice straw

Rice straw was selected for the study, which was obtained from a farm located at Baramati, Maharashtra. As mentioned earlier, rice straw contains complex polymers, such as cellulose, hemicellulose and lignin, which are susceptible to microbial degradation. Thus, these are the primary target polymers for hydrolytic-enzyme producing bacteria, which play a role in destabilization of the structure of stubble. In order to increase the surface area of the stubble for the microbial action, the rice straw was mechanically ground using a mixer grinder to obtain coarse particles. The degradation experiment was run in presence of excess water.

#### 2.4.2 Microbial decomposition of stubble

The bacterial culture was grown in sterile nutrient broth as described previously. A medium was prepared in 250 ml conical flask containing 100 ml of distilled water, 10 g dried stubble matter and ammonium sulfate (0.2 g) at pH 7.0. The medium was sterilized and separately inoculated with 1 ml of suspension (10^8^ CFU/ml) of CMB10 and SFMB9. A trial flask with co-inoculation of both cultures was also prepared by inoculating 0.5 ml of each of the suspensions. The sets of uninoculated control (stubble medium) were also run parallel. The flasks of each treatment were labelled as 0 day, 15^th^ day, 30^th^ day and 45^th^ day. All the treatment flasks were incubated at 30 °C for 15, 30 and 45 days with periodic shaking. All the trials were repeated three times.

*Dry weight analysis -* The sample treatment flasks collected at regular time intervals were analyzed for dry weight. The stubble samples were filtered and dried at 80 °C till constant weight was obtained. The degradation percentage was calculated based on the dry weight of the samples.

*SEM analysis* – The morphological characterization of rice straw (dried powder) was further analyzed by Scanning Electron Microscopy (SEM) to note the structural changes in stubble due to microbial degradation [14]. The analysis was carried out at National Chemical Laboratory, Pune, India.

*Assay for amylase, pectinase, xylanase and cellulase –* The filtrate from each flask collected at different time intervals was analyzed for the enzyme activity. An appropriately diluted 250 μl of supernatant was added to 250 μl of substrate. Substrates such as 1 % starch, pectin, xylan, and CMC were used for the determination of amylase, pectinase, xylanase and cellulase activity [15]. Briefly, the reaction mixture was incubated at 30 °C for 10 min. The reaction was stopped by the addition of 500 μl of DNSA reagent and incubated in a boiling water bath for 10 min. The tubes were cooled and diluted to 4 ml with distilled water followed by measuring absorbance at 550 nm. One unit of enzyme activity was defined as the amount of enzyme releasing 1 μmol of reducing sugar (glucose) per ml per min under assay conditions.

*Analysis of C/N ratio –* The sample was collected periodically during stubble degradation and total organic carbon was determined by loss of weight after ignition at 550 °C for 8 h as described by Chefetz et al. [16]. The total organic nitrogen was determined by N_2_ analyzer (Kelplus nitrogen estimation system) by Kjeldahl method.

### 2.5 Effect of stubble degradation product on plant growth

The slurry (stubble degradation product) obtained from the above degradation experiment was further tested for its effect on the growth of *Glycine* *max* (JS-335). Garden soil was dried under sunlight followed by removal of debris, breaking of soil aggregations and sterilization. The experimental treatment pots were filled with 1 Kg of soil. The treatments were divided into four groups: CMB10, SFMB9, CMB10+SFMB9 and uninoculated control (UC). Each treatment group was further subdivided according to days post inoculation i.e., 15, 30 and 45. For example, for CMB10, treatment subgroups were CMB10-15, CMB10-30 and CMB10-45; for SFMB9, SFMB9-15, SFMB9-30 and SFMB9-45; for CMB10+SFMB9, CMB10+SFMB9-15, CMB10+SFMB9-30 and CMB10+SFMB9-45; for uninoculated control, UC-15, UC-30, UC-45. All the trials were conducted in triplicate and each pot served as a replicate of the representative treatment group. The slurry obtained at different time intervals from inoculated and uninoculated control was added to the respective treatment pot. Each pot received four healthy seeds of *G. max*. The pots were labelled and placed in a net-house where pots were watered twice a day. After 20 days of incubation, the plants were harvested by uprooting them. These plants were then analyzed for root and shoot length, and chlorophyll content of leaves [17].

### 2.6 Statistical analysis

The data on the effect of stubble compost on plant growth was analyzed for the significant difference by using one-way ANOVA and Post Hoc Test (Tukey HSD) using SPSS 18. A 95 % confidence level was used for the analysis so that *P* ≤ 0.05 were considered to be statistically significant.

## 3 Results

### 3.1 Screening for cellulolytic bacteria and determination of their diverse enzymatic potential

In all, 120 bacterial isolates were recovered from five different sources viz., garden soil, compost, agriculture soil and wastewater. Out of 63 cellulolytic bacterial isolates, 15 were obtained from wastewater samples, 18 from compost, 8 from vegetable waste dump soil, 13 from garden soil and 9 from agriculture soil samples. All these isolates were further screened for other hydrolytic enzyme activity viz., pectinase, amylase, xylanase and protease. The potent isolates were selected based on their enzymatic potential. In comparison, isolate CMB10 and SFMB9 were found to be the most potent hydrolytic enzyme producers with the ability to grow on lignin containing medium, indicating their lignolytic potential.

### 3.2 Characterization of potent polymer degraders

Two potent bacterial isolates, SFMB9 and CMB10, were characterized by a phenotypic approach. Both the isolates were rod-shaped, Gram-negative, non-motile and capsulated. The colonies of SFMB9 were milky white, with entire margin, opaque and mucoid, whereas, CMB10 colonies were white, with entire margin, opaque and sticky consistency. In molecular studies following BLAST search of 16S rDNA nucleotide sequences at NCBI GenBank database, the isolates, SFMB9 and CMB10, showed more than 99 % similarity with *Agrobacterium pusense* and *Olivibacter oleidegradans,* respectively. A phylogenetic tree was constructed using representative sequences of 16S rDNA obtained from the NCBI GenBank database (Fig. 1). Both the sequences were submitted at the NCBI GenBank database and the accession numbers obtained for SFMB9 and CMB10 are OQ165320 and OQ167783, respectively.

**Fig. 1:**
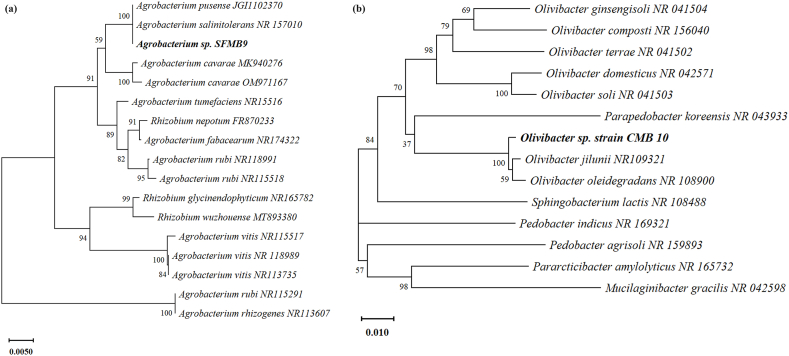
Phylogenetic relationship of *Agrobacterium pusense* SFMB9 (a) and *Olivibacter oleidegradans* CMB10 (b) based on 16S rDNA sequence data. The evolutionary history was derived using the Neighbour-Joining method. The percentage of replicate trees in which the associated taxa clustered together in the bootstrap test (1000 replicates) are shown next to the branches. The tree is drawn to scale, with branch lengths in the same units as those of the evolutionary distances used to infer the phylogenetic tree. The evolutionary distances were computed using the Kimura 2-parameter method and are in the units of the number of base substitutions per site. Evolutionary analyses were conducted in MEGA X.

### 3.3 In-vitro rice stubble degradation studies

**Fig. 2:**
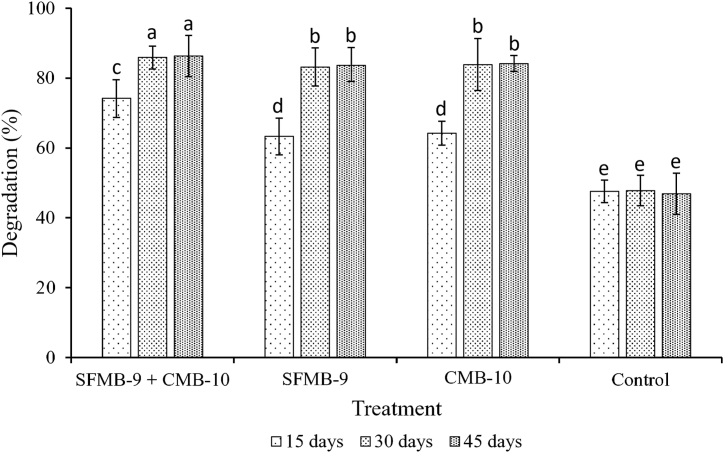
Microbial degradation of rice stubble. The trials include inoculation of rice stubble medium with SFMB9, CMB10, co-inoculation of SFMB9 and CMB10, and uninoculated control. Degradation (%) was calculated on the basis of dry weight of samples at 15^th^, 30^th^ and 45^th^ day. The same letters on the columns indicate no significant difference between treatments according to Tukey's HSD for significant difference (*P* < 0.05).

SFMB9 and CMB10 were tested for the degradation of rice stubble under in-vitro conditions. The cultures were used separately as well as in combination and the treatment period was from 0 days to 45 days. The treatment flasks were harvested for the determination of dry weight followed by calculation of degradation percentage on 0, 15^th^, 30^th^ and 45^th^ day. In the case of combined treatment of bacterial cultures, the degradation (%) was recorded on 15^th^, 30^th^ and 45^th^ day as 74.2, 85.9 and 86.3 %, respectively, which were highest in comparison to individual bacterial treatments. On 45^th^ day, in the case of SFMB9 and CMB10 treatments, the degradation values were 83.7 and 84.2 %, respectively. As shown in Fig. 2, a combination of bacterial treatments was more effective than single culture treatments (*P* < 0.05) on 30^th^ and 45^th^ day post-inoculation. However, the single culture treatements with SFMB9 and CMB10 were equally effective (*P* > 0.05). All the inoculated treatments showed significant degradation of rice stubble on all days post-inoculation in comparison to the uninoculated control (*P* < 0.05).

**Fig. 3:**
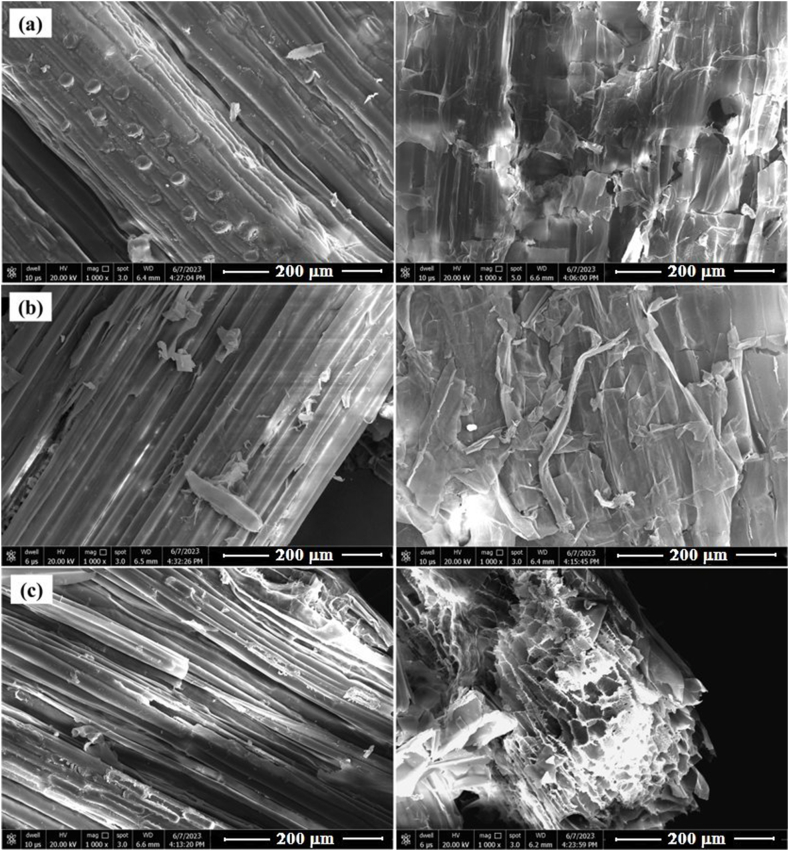
Scanning electron microscope images of untreated and treated stubble after 45 days of incubation. (a) (right side), Stubble was treated with *Olivibacter oleidegradans* CMB10; (b) (right side), Stubble was treated with *Agrobacterium pusense* SFMB9; (c) (right side), Stubble was treated with both the cultures. All left side images of (a), (b) and (c) represent untreated controls of respective trials.

The degradation of rice stubble was also confirmed by SEM analysis. SEM images of untreated and treated stubble after 45 days of incubation are shown in Fig. 3. All right-side images are of rice straw treated with bacterial cultures where it is distorted. Also, the separation from initially connected structures can be noted. However, in the case of untreated control, a stable structure and highly ordered fibrils can be observed.

**Fig. 4:**
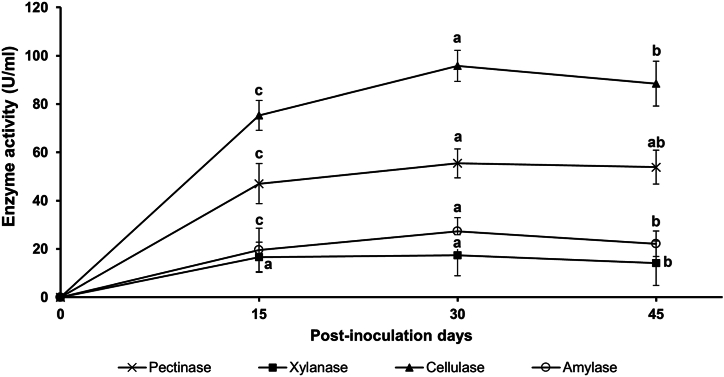
Hydrolytic enzyme activity in co-inoculated trial after a treatment period of 45 days, where, the stubble was treated with co-inoculation of SFBM9 and CMB10 cultures. The same letters on each data point corresponding to each enzyme activity indicate no significant difference (*P* < 0.05) between different time intervals. Tukey's Post Hoc Test analyzed data for significant difference.

The samples periodically collected during the degradation studies were analyzed for the hydrolytic enzyme activity. As shown in Fig. 4, in co-inoculated trial, the activity of cellulase was at highest (95.77 U/ml) on 30^th^ day post-inoculation, which was significantly higher in comparison to 15^th^ and 45^th^ day samples (*P* < 0.05). The activity of all other enzymes was less as compared with cellulase, however, the activity of all other enzymes was higher at 30^th^ day. Elevated pectinase activity (55.54 U/ml) was also noted followed by cellulase. There was a decline in enzyme activity after the 30^th^ day post-inoculation. The activity of xylanase (17.33 U/ml) and amylase (27.33 U/ml) was the lowest among all the enzymes.

**Fig. 5:**
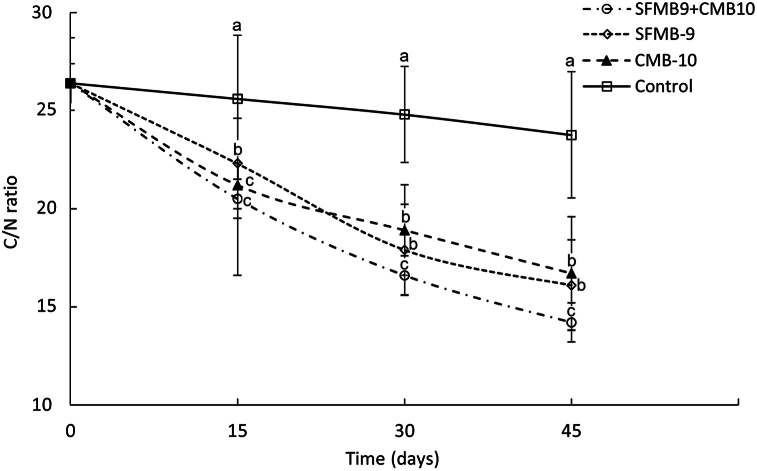
The content of C/N ratio of rice straw during 45 days of composting. Bacterial treatments include SFMB9, CMB10, co-inoculation, and an uninoculated control. The same letters on each data point on the respective day indicate no significant difference between treatments, where *P* < 0.05 is considered significant concerning Tukey's HSD.

All the samples collected periodically were also analyzed for total carbon and total nitrogen for the calculation of C/N ratio, an indicator of maturity degradation. In all inoculated trials, the C/N ratio decreased, however, the highest decrease was recorded in the case of co-inoculated trials (SFMB-9 + CMB-10). Similarly, total nitrogen content during the composting period increased in the case of all the inoculated trials. As a result, as shown in Fig. 5, the C/N ratio decreased during a period of 45 days except in the uninoculated trial from 26.4 to 14.2. However, the decline was significant in the case of co-inoculated trial (*P* < 0.05).

### 3.4 Effect of stubble degradation product on the growth of *Glycin max*

**Fig. 6:**
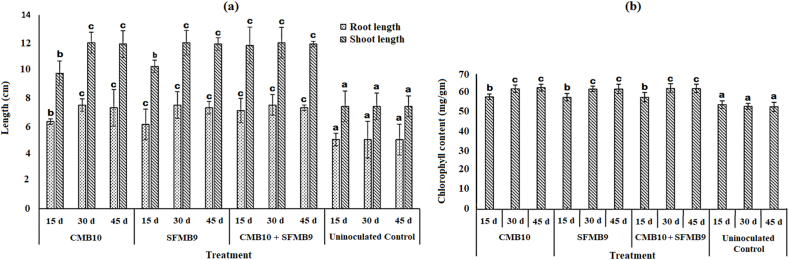
Effect of stubble compost on the growth of *Glycinmax* in terms of root and shoot length (a) and chlorophyll content (b). The stubble compost prepared from CMB10, SFMB9 and co-inoculation followed by incubation for 15, 30 and 45 days were tested separately along with an uninoculated control. The experiment was repeated three times. The same letters on the columns with the same fill indicate no significant difference as determined by Tukey's Post Hoc Test where *P* ≤ 0.05 is considered significant.

Further, the degradation product obtained following bacterial treatments was tested for plant growth-promoting activity on *G. max*. This compost obtained through bacterial treatments on 15^th^, 30^th^ and 45^th^ day showed improvement in root and shoot length in comparison to uninoculated control (Fig. 6a). The compost obtained from bacterial treatments on 30^th^ and 45^th^ day was equally effective in promoting root and shoot length (*P* > 0.05). Also, there was an improvement in the chlorophyll content of plants treated with bacterial compost (Fig. 6b). The bacterial compost obtained on 30^th^ and 45^th^ day showed an increase in the chlorophyll content of treated plants (*P* > 0.05).

## 4 Discussion

Rice straw is one of the important agricultural wastes generated every year in huge amounts in the world, however, its disposal is a problem [18,19]. Intentionally, most farmers use burning practice for its disposal which is considered as easiest and most economical method. This may be due to the availability of less period between harvesting and cultivation of new crops, unavailability of labour and lack of awareness about management methods. However, burning of stubble is associated with harm to the environment and human health. Many workers suggested the valorization of this waste to compost fertilizer using different types of microorganisms such as bacteria [20], actinomycetes [21], and fungi [22]. This compost can be further utilized for plant growth promotion in different crops. These microbes have the unique ability of synthesis of plant cell wall degrading enzymes such as cellulase, amylase, ligninase, pectinase and xylanase. However, cellulase is one of the key enzymes in the degradation of lignocellulolytic wastes such as rice stubble [23]. In this view, in the present investigation, cellulolytic bacteria were screened for the synthesis of different hydrolytic enzymes. Two potent bacterial isolates with the ability of synthesis of xylanase, amylase and pectinase were selected for the degradation studies. These bacteria were capable of growing on lignin-containing media too. Phylogenetic analysis based on 16S rDNA sequences of SFMB9 and CMB10 revealed the close affiliation of these isolates with the *Agrobacterium* and *Olivibacter* genus, respectively.

Genus *Olivibacter* is one of the unique members of the family Sphingobacteriaceae, commonly isolated from water and soil. A few strains of *Olivibacter* have been reported from different pesticide and hydrocarbon-contaminated soil samples. Oil-degrading *O. oleidegradans* has been isolated from hydrocarbon contaminated soil [24]. Ntougias et al. [25] reported the isolation of *O. sitiensis* from olive-oil mill wastes and Chen et al. [26] isolated *O. jilunii* from DDT contaminated soil. *O. ginsenosidimutans* [27] and *O. composti* [28] have been reported from the compost samples. Thus, the literature on *Olivibacter* strains is scarce. During the current investigation, *Olivibacter* sp. CMB10 was isolated from the compost sample and following 16S rRNA gene sequencing and analysis *O. oleidegradans* was the closest neighbour after BLAST analysis at NCBI GenBank. Another isolate of the current investigation, *Agrobacterium* sp. SFMB10, was isolated from a rhizospheric soil sample that showed a close match with *A. pusense* following BLAST analysis. Genus *Agrobacterium* is one of members of Rhizobiaceae family that are commonly found in soil associated with plant roots. *Agrobacterium* sp. are known for its various applications in agriculture and the environment. *Agrobacterium* strain JAS1 has been described for the degradation of azo dyes by Kaur et al. [29]. Few are responsible for the formation of tumors on the stems, and roots of plants. Due to this ability, *A. tumefaciens* has been widely used as a natural tools for the genetic transfers in genetic engineering for the transformation of plants of agricultural interest [30]. However, a few strains of *A. tumefaciens* have been also used for plant growth promotion [31]. *A. pusense* strain NRCPB10T has been isolated from the rhizosphere of chickpea plants that did not nodulate chickpea plants or induce tumors in tobacco plants [32]. Similarly, there are reports on the isolation and characterization of *A. pusense* from bean root nodules [33]. Kaur et al. [34] reported the isolation of exopolysaccharide producing *A. pusense* that enhances plant growth. In a recent investigation, Chang et al. [35] explored the phosphate solubilizing potential of *A*. *deltaense*. However, there are no reports on the use of *Agrobacterium* sp. in stubble degradation so far. Thus, the role of *Olivibacter* sp. and *Agrobacterium* sp. in connection with stubble degradation has been described here for the first time.

Attempts to degrade agricultural wastes with bacteria have been investigated with the use of single strains, dual compatible strains, and complex microcosms comprising multiple strains of fungi and bacteria. Several investigators have emphasized the potential usefulness of mixed culture in accelerating the biodegradation efficiency of different agricultural wastes [36]. In fungal degradation studies, the use of a consortium also proved to be the best system compared to single culture studies. Similar rice stubble degradation studies with mixed cellulolytic fungal isolates such as *Cheatomium globusum*, *Penicillium oxalicum, Trichoderma reesei* and *Aspergillus niger* also resulted into a significant increase in the rice straw degradation rate as compared to the counter part single-strain culture [37]. Sruthy et al. [38] have described the use of fungal consortium constructed with *Asp. terreus*, *Asp. fumigatus* and *Alternaria* sp. for the management of rice stubble. The use of a single bacterial culture usually resulted in a relatively low decomposition rate. To increase the decomposition capability of the bacteria, a rational mixed culture of selected bacteria was employed [39]. Yang et al. [40], confirm the use of microbial combination culture consortium over a single culture for the management of agriculture residues. Ban et al. [41] have quoted the importance of bacterial consortia in the degradation of paddy straw waste that includes the species of *Pseudomonas*, *Comamonas*, *Fibrobacter*, *Sphingobacterium*, *Ruminiclostridium*, and *Ruminococcus.* Likewise, Sharker [42] isolated and characterized two *Bacillus* and a *Citrobacter* isolate with robust cellulose-degrading capabilities from cow rumen and forest soil to unveil a novel enzymatic approach involving these strains for efficient rice straw biomass deconstruction. In a recent study, a consortium of cellulolytic strains of *B. amyloliquefaciens* and *B. velezensis* was constructed and used in the degradation of spent mushroom substrate [43]. Poszytek et al. [44] have also demonstrated the effectiveness of a microbial consortium with high cellulolytic activity in the management of maize silage for the production of biogas. The consortium was constructed by using strains of *Bacillus* sp., *Providencia* sp. and *Ochrobactrum* sp. Along similar lines, the present investigation also demonstrated the effectiveness of the use of co-culture approach using *Agrobacterium* sp. SFMB9 and *Olivibacter* sp. CMB10 in the composting of rice stubble.

The bio-compost generated through the microbial mineralization process may be incorporated into the soil to improve its nutritional condition and plant growth [10]. Several studies have demonstrated the enhancement in the growth and yield of vegetables, fruit and grain crops [[45], [46], [47]]. All such studies have indicated the benefits of using compost in agriculture. The reports of the present investigation also support the positive impact of stubble compost on the growth of *G*. *max* through improvement in root and shoot length and increase in chlorophyll content. The degradation of rice stubble by microorganisms to simple organic compounds is indicated by several parameters. SEM is often used for the visual evaluation of degradation that reveals the structural modifications generated due to microbial decomposition [14,48]. Long et al. [43] recently demonstrated the destabilization of the lignocellulosic structure of spent mushroom substrate using SEM. The study found that, in its raw waste form, the surface morphology of the cellulosic waste (spent mushroom substrate) exhibited a smooth and compact surface with better integrity. However, after 20 days of microbial consortium degradation treatment, the surface microstructure of the waste showed noticeable changes. During the present investigation, SEM revealed the difference between the structural appearance of treated and untreated rice stubble where after microbial treatment the stubble structure was severely damaged. Also, the elevated levels of cellulase, pectinase and xylanase in test treatment indicated the potential for degradation of rice stubble by SFMB9 and CMB10 isolates. Determination of the C/N ratio is another parameter used to judge the rate of decomposition during composting process. The decomposition of organic residues by microbes results in the loss of the total carbon content of the matter [49]. During such microbial degradation processes, chemoorganotrophic microbes utilize organic compounds as a source of carbon and energy resulting in the formation of CO_2_ [50]. The C/N ratio thus declines during microbial degradation [51]. As determined during the present investigation, C/N ratio of the stubble residues decreased following microbial decomposition for 45 days. The stubble compost obtained by single or co-inoculation of bacterial cultures was considered mature as a value below 20 indicates the maturity of compost [[52], [53], [54]].

The effectiveness of SFMB9 and CMB10 isolates in different combinations was tested in this study for efficient degradation of rice stubble. Overall, the results of this study demonstrate that the extracellular enzyme activity of these bacterial isolates in mixed culture as co-inoculants may be exploited for valorization of rice stubble under field conditions to enhance the rates of degradation of these types of agriculture residues to recycle carbon, nitrogen and other nutrients in the soil, thereby discouraging the farmers from burning them in the fields. However, in the present investigation, degradation was carried out at the flask level under controlled conditions. Modifications to the process will be necessary when scaling up to the field level. Further, the release of reducing sugars by the action of extracellular enzymes of these isolates, particularly as co-inoculants, suggests that these cultures may be commercially exploited by agro-waste industries for the production of bio-ethanol using rice stubble. Additionally, this mixed culture of cellulolytic bacteria can be employed as robust biocatalysts in microbial fuel cells (MFCs) to generate electricity by utilizing cellulose present in rice stubble as an organic substrate [55]. More recently, rice straw has been also used both as a source and carbon substrate for the isolation and production of biodegradable polyester polyhydroxyalkanoate (PHA) bacteria [56]. Apart from this, another suitable approach for the biotreatment of rice stubble can be developed by employing mixed culture of anaerobic bacteria for efficient digestion of these agriculture waste biomass and thereby effectively enhancing the biogas yield [57]. Sangwan and Deswal [58] explored and highlighted the various microbial mediated in-situ sustainable management strategies that can be employed for the strategic biodegradation of rice stubble. It is worth mentioning that the cooperative action of cellulolytic microbes provides the basis for the degradation of lignocellulosic wastes in nature and is considered a potent approach for developing efficient microbial consortiums for biotechnological applications, such as processing of agricultural wastes like rice stubble. In this sense, metagenomic approaches might provide an answer for identifying promising microorganisms and unveiling a range of enzymes involved in the efficient hydrolysis of rice straw.

## 5 Conclusion

Composting organic residue such as rice stubble is an interesting approach for the recycling of waste, as such compost obtained following microbial degradation is used as organic fertilizer in agriculture. In order to biologically hydrolyze rice stubble under field conditions or in the laboratory, a set of unique enzyme combinations is a prerequisite. In present investigation, a combined culture treatment of rice stubble caused 86.3 % of degradation on 45^th^ day post-inoculation, which was highest in comparison to individual bacterial treatments. However, both the singly inoculated treatments showed significant degradation of rice stubble on all days post-inoculation in comparison to the uninoculated control. For the first time, this work describes the management of rice stubble and its valorization for plant growth promotion using a bacterial consortium comprising *Olivibacter* sp., isolated from compost, and *Agrobacterium* sp., isolated from the rhizosphere. These isolates have the potential to synthesize multiple enzymes that aid in the mineralization of organic residues. In co-inoculated trial, the cellulase activity was at highest (95.77 U/ml) on 30^th^ day post-inoculation. The level of pectinase was also found elevated (55.54 U/ml) followed by xylanase (17.33 U/ml) and amylase (27.33 U/ml). This investigation conducted on the biodegradation of rice stubble showed the high potential for using a mixed culture of *Olivibacter* sp. and *Agrobacterium* sp. for effective straight-forward and inexpensive degradation of rice straw in an open environment to recycle carbon, nitrogen and minerals in the soil. In all, the study suggests the use of inexpensive biological approach that enhances the rate of biodegradation of rice stubble in the field that may discourage the farmers to burn it in the field and save the environment by managing this agricultural waste. These aggregative results suggested a novel model for rice straw degradation utilizing hydrolytic enzymes of the consortium, revealing superior efficacy compared to individual strains, and advancing cost-effective, affordable, and sustainable green technology.

## CRediT authorship contribution statement

**Milind Gajbhiye:** Writing – review & editing, Writing – original draft, Project administration, Formal analysis. **Sushmita Patil:** Investigation. **Sagar Awate:** Investigation. **Supriya Kokare:** Investigation. **Siddharth Terdale:** Investigation. **Manish Kumar Dubey:** Writing – review & editing, Supervision.

## Data availability

No external data was utilized in the research described in the article.

## Ethics declaration

This study did not require review or approval by an ethics committee or informed consent because the article did not involve any direct experimentation or studies on humans or animals.

## Funding

The research did not receive any funding.

## Declaration of competing interest

The authors declare that they have no known competing financial interests or personal relationships that could have appeared to influence the work reported in this paper.

## Figures and Tables

**figs1:**
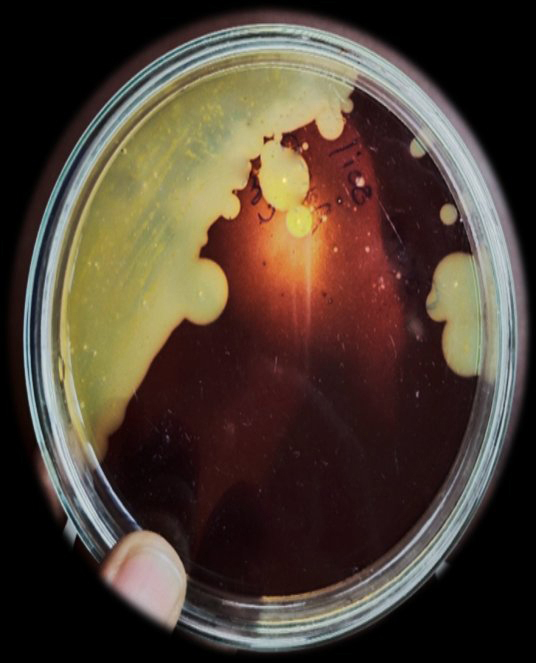


**figs2:**