# Supplementary material for: Valorization of rice stubble through biodegradation using hydrolytic enzyme-producing Olivibacter oleidegradans CMB10 and Agrobacterium pusense SFMB9
Source: Heliyon. 2025 Jan 17;11(2):e42094. doi: 10.1016/j.heliyon.2025.e42094 (PMC11804540; doi:10.1016/j.heliyon.2025.e42094)
Supplement: Multimedia component 1 [file mmc1.docx]

Table 1. Relative enzyme activity of selected bacterial isolates

| Sr. No. | Isolates Code | Relative enzyme activity^*^ | | | | |
| --- | --- | --- | --- | --- | --- | --- |
|  |  | Cellulase | Amylase | Protease | Pectinase | Xylanase |
| 1 | GS-11 | 0.5 | 0.3 | 0.3 | 0.3 | - |
| 2 | GS-20 | 0.5 | 0.3 | - | 0.3 | - |
| 3 | CMB-10 | 0.86 | 0.5 | 0.33 | 0.66 | 0.5 |
| 4 | CMB-30 | 0.6 | 0.3 | - | 0.5 | 0.33 |
| 5 | CMB-34 | 0.6 | 0.3 | 0.33 | 0.5 | - |
| 6 | CMB-41 | 0.6 | 0.3 | - | 0.5 | - |
| 7 | SFMB-2 | 0.5 | 0.3 | - | 0.3 | - |
| 8 | SFMB-9 | 0.8 | 0.5 | 0.33 | 0.6 | 0.5 |
| 9 | SFMB-19 | 0.6 | 0.3 | - | 0.5 | 0.33 |
| 10 | SFMB-21 | 0.74 | 0.5 | 0.33 | 0.6 | 0.5 |
| 11 | SFMB-22 | 0.6 | 0.3 | 0.33 | 0.5 | - |
| 12 | CM-3 | 0.5 | 0.2 | - | 0.5 | - |
| 13 | CM-5 | 0.5 | 0.3 | - | 0.5 | - |
| 14 | CM-9 | 0.5 | 0.3 | - | 0.5 | - |
| 15 | CM-11 | 0.5 | 0.3 | - | 0.5 | - |
| 16 | WOB-12 | 0.6 | 0.3 | - | 0.5 | - |
| 17 | WOB-14 | 0.5 | 0.3 | - | 0.3 | - |
| 18 | WOB-18 | 0.5 | 0.3 | - | 0.3 | - |
| 19 | WOB-18 | 0.5 | 0.3 | - | 0.3 | - |
| 20 | WOB-18 | 0.5 | 0.3 | - | 0.3 | - |

$$*Relative enzyme activity =\frac{Colony diameter including clearance-Colony diameter}{Colony diameter including clearance}$$
