# Supplementary material for: Valorization of rice stubble through biodegradation using hydrolytic enzyme-producing Olivibacter oleidegradans CMB10 and Agrobacterium pusense SFMB9
Source: Heliyon. 2025 Jan 17;11(2):e42094. doi: 10.1016/j.heliyon.2025.e42094 (PMC11804540; doi:10.1016/j.heliyon.2025.e42094)
Supplement: Multimedia component 2 [file mmc2.docx]

Table 2.1 Colony characters of bacterial isolates

| Isolate code | 16S rDNA sequencing identity | Colony characters | | | | | | |
| --- | --- | --- | --- | --- | --- | --- | --- | --- |
|  |  | Size | Shape | Colour | Consistency | Margin | Elevation | Opacity |
| CMB-10 | *Olivibacter oleidegradans* | 2mm | Circular | Off white | Smooth | Entire | Convex | Opaque |
| SFMB-9 | *Agrobacterium* *pusense* | 2mm | Circular | Milky white | Smooth | Entire | Convex | Opaque |

Table 2.2 Morphological features of bacterial isolates

| Isolate code | Shape | Motility | Gram character | Arrangement |
| --- | --- | --- | --- | --- |
| CMB-10 | Rod | Non-motile | Gram-negative | Singular |
| SFMB-9 | Rod | Non-motile | Gram-negative | Singular |
